# Molecular Orientations of Delayed Fluorescent Emitters in a Series of Carbazole-Based Host Materials

**DOI:** 10.3389/fchem.2020.00427

**Published:** 2020-05-25

**Authors:** Hisahiro Sasabe, Yuki Chikayasu, Satoru Ohisa, Hiroki Arai, Tatsuya Ohsawa, Ryutaro Komatsu, Yuichiro Watanabe, Daisuke Yokoyama, Junji Kido

**Affiliations:** ^1^Research Center for Organic Electronics (ROEL), Yamagata University, Yamagata, Japan; ^2^Frontier Center for Organic Materials (FROM), Yamagata University, Yamagata, Japan; ^3^Department of Organic Materials Science, Graduate School of Organic Materials Science, Yamagata University, Yamagata, Japan

**Keywords:** delayed fluorescence, molecular orientation, light out-coupling efficiency, carbazoles, weak hydrogen bonds

## Abstract

Molecular orientation is one of the most crucial factors to boost the efficiency of organic light-emitting devices. However, active control of molecular orientation of the emitter molecule by the host molecule is rarely realized so far, and the underlying mechanism is under discussion. Here, we systematically investigated the molecular orientations of thermally activated delayed fluorescence (TADF) emitters in a series of carbazole-based host materials. Enhanced horizontal orientation of the TADF emitters was achieved. The degree of enhancement observed was dependent on the host material used. Consequently, our results indicate that π-π stacking, CH/n (n = O, N) weak hydrogen bonds, and multiple CH/π contacts greatly induce horizontal orientation of the TADF emitters in addition to the molecular shape anisotropy. Finally, we fabricated TADF-based organic light-emitting devices with an external quantum efficiency (η_ext_) of 26% using an emission layer with horizontal orientation ratio (Θ) of 79%, which is higher than that of an almost randomly oriented emission layer with Θ of 62% (η_ext_ = 22%).

## Introduction

A series of fluorescent emitters exhibiting significant delayed fluorescence, so-called thermally activated delayed fluorescent (TADF) emitters, has attracted much attention due to its potential usefulness in high-performance organic light-emitting devices (OLEDs) that can realize an internal quantum efficiency (η_int_) of 100% (Uoyama et al., 2012; Sasabe and Kido, 2013; Adachi, 2014; Kaji et al., 2015; Lin et al., 2016; Im et al., 2017; Wong and Zysman-Colman, 2017; Yang et al., 2017; Komatsu et al., 2018). In principle, TADF emitters consist of electron-donor (D) and electron-acceptor (A) moieties realizing efficient intramolecular charge transfer (ICT). The connection between D and A moieties is generally accompanied with a small overlap in the frontier molecular orbital (FMO) between the highest occupied molecular orbital (HOMO) and the lowest unoccupied molecular orbital (LUMO), in other words, a small energy difference between singlet and triplet energies (Δ*E*_ST_). Recent rapid development in TADF emitters enables OLEDs to achieve an external quantum efficiency (η_ext_) over 30% (Kaji et al., 2015; Komino et al., 2016; Lin et al., 2016; Liu et al., 2017; Rajamalli et al., 2017; Wu et al., 2018; Ahn et al., 2019; Kondo et al., 2019). To obtain such a high-performance in OLEDs, horizontal orientation of the emission dipole moment (EDM) is absolutely essential. Perfect horizontal orientation of the EDM has been reported to boost OLED

efficiency up to 150% compared to random EDM orientation (Frischeisen et al., 2011; Yokoyama, 2011; Schmidt et al., 2017; Kim and Kim, 2018; Watanabe et al., 2019b). However, the horizontal orientation ratio (Θ) of most TADF emitters used in OLEDs realizing η_ext_ over 30% is reportedly only around 80%, which is far behind perfect horizontal orientation (Θ = 100%). In general, a guest TADF emitter is dispersed into a host material to reduce concentration quenching, and to maintain carrier balance in the emission layer (EML). The proportion of guest molecule is commonly much smaller than that of host molecule. Thus, the non-covalent interactions between host and guest molecules should be one of the important factors that controls the molecular orientation.

In an early stage of study on molecular orientation in OLEDs, Yokoyama used variable-angle spectroscopic ellipsometry (VASE) measurements to reveal the anisotropy of the molecular shape, such as in planar and linear structures, is essential to realize horizontal orientation of a series of triphenylamine and carbazole based-fluorescent molecules, even in randomly oriented host molecules (Yokoyama et al., 2009). However, unlike first-generation fluorescent molecules, most TADF molecules do not have a planar structure but instead a winding chemical structure in needed to realize a small overlap of the frontier molecular orbitals in order to achieve efficient reverse intersystem crossing (RISC). Therefore, in addition to the molecular shape anisotropy, advanced strategies to actively use non-covalent interactions to realize enhanced horizontal orientation are highly desired.

In 2016, Lin and Wong reported three types of sky-blue TADF emitters using triphenyltriazine as an acceptor and substituted acridines as a donor unit (Lin et al., 2016). Among these, **SpiroAc-TRZ** with spirobiphenyl unit showed a photoluminescent quantum yield (η_PL_) of 100%, and a high Θ value of 83% in **mCPCN** host. They indicated that this high Θ value of **SpiroAc-TRZ** is attributed to the overall planar and balanced/symmetrical structure. The corresponding OLED exhibited an extremely high η_ext_ of nearly 37%. In the same year, Komino and Adachi reported the complete horizontal orientation of a linear-shaped TADF emitter named **Cis-BOX2** in a randomly oriented host matrix at the temperature of 200 K (Komino et al., 2016). The horizontal orientation ratio of **Cis-BOX2** depended on the deposition temperature and the type of host matrix. A **Cis-BOX2**-based OLED showed a very high η_ext_ of 33%.

**Figure 1:**
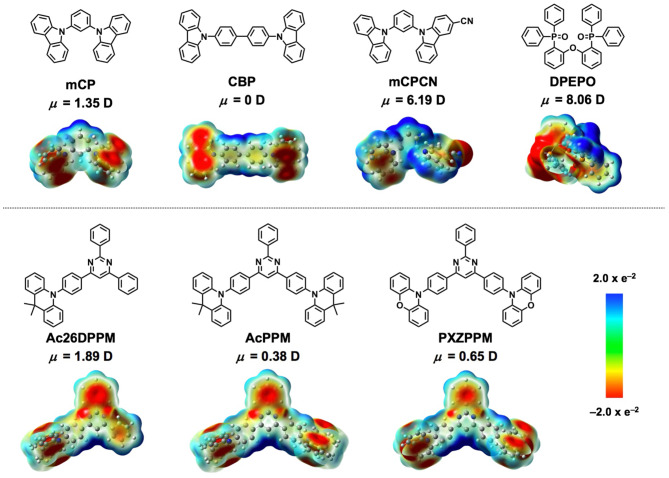
Chemical structures, dipole moments, and electronic surface potentials (ESP) of host molecules **(top)** and TADF emitters **(bottom)** used in this study.

As mentioned above, although a high Θ value of up to 100% at 200 K has been reported, the underlying mechanism to realize horizontal orientation is still under discussion at this stage (Yokoyama, 2011; Mayr and Brütting, 2015; Moon et al., 2015; Shibata et al., 2015; Friederich et al., 2017; Schmidt et al., 2017; Gujral et al., 2018; Kim and Kim, 2018; Lee et al., 2018; Pal et al., 2018; Watanabe et al., 2019b). In order to deepen insights into the underlying mechanism, we wish to report a systematic investigation on the molecular orientations of TADF molecules in a series of carbazole-based host materials. To reveal the structure–property relationship for horizontal orientation, a series of TADF emitters, which we can systematically change the parameters one by one, should be necessary. In this context, we have already developed a series of pyrimidine-based TADF emitters with different donor group(s) (Figure 1) (Komatsu et al., 2016a,b; Nakao et al., 2017). By comparing these three molecules, we can obtain insight into the effects of two factors: (i) donor number, which reflects the molecular shape anisotropy in these comparisons, and (ii) donor structure. Consequently, we achieved enhanced Θ values of TADF emitters and the degree of enhancement depended on the host material used. Consequently, our results indicate that increased π-π stacking, CH/n (n = O, N) weak hydrogen bond (H-bond) interaction, and multiple CH/π contacts greatly induce horizontal orientation of TADF emitters in addition to the molecular shape anisotropy. Finally, we fabricated a TADF-based OLED with η_ext_ of 26% using an emission layer (EML) with a Θ value of 79%, which is greater than that of an almost randomly oriented EML with a Θ value of 62% (η_ext_ = 22%).

## Results and Discussion

### Selection of Carbazole-Based Host Molecules and Phosphine-Oxide Host Molecules

**Figure 2:**
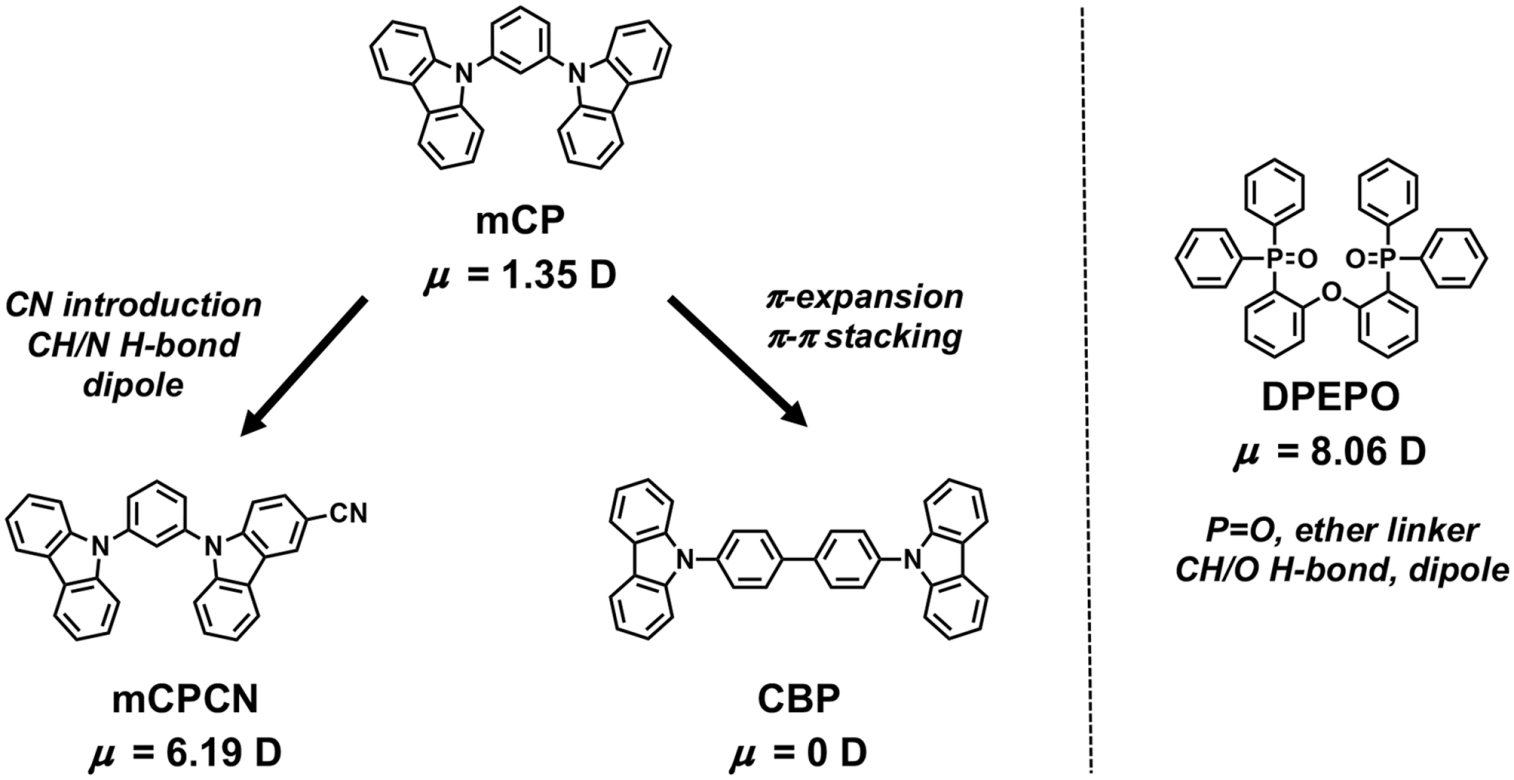
Intermolecular interactions introduced when comparing the host materials to the mCP benchmark.

Carbazole derivatives are one of the most popular host materials in OLEDs. Therefore, insights into carbazole-based host materials are considered to be highly valuable. To identify the underlying mechanisms of horizontal orientation, we selected four types of host materials to study and sequentially changed their chemical structures (Figure 1 and Table 1). **mCP** was used as the benchmark material. Extending its π-conjugation, results in **CBP**, which can be used to evaluate the effect of π-conjugation or π-π stacking when compared to **mCP**. The introduction of CN substituent into **mCP** forms **mCPCN** (Lin et al., 2012). Using this molecule, we can validate the effect of CN substituent, such as the dipole/dipole interaction and CH/N weak H-bonds. A phosphine-oxide host material, **DPEPO** (Han et al., 2011) was used to investigate the effect of the dipole moment and CH/O weak H-bonds of P=O substituents (Figure 2). Note that all the host materials used in this study showed random orientation. DFT calculations were performed at B3LYP 6-31G(d) level to evaluate the dipole moment and visualize the electronic surface potential (ESP), as shown in Figure 1 and Figure S1 (in supporting information). Among the host materials, **mCP** and **CBP** had small dipole moment <1.5 D, while **mCPCN** with a cyano group and **DPEPO** with phosphine oxide groups possessed a large dipole moment >6.0 D. ESP exhibited strong negative charge (red color) on the π-plane of the carbazole and on the electron-withdrawing groups of CN and P=O. The hydrogen (H) atoms on the aromatic rings had positive charge (blue color).

**Table 1:** Thermal and optical properties of the host molecules.

**Compound**	**Mw (g/mol)**	**$T_{g}^{a}$/$T_{\text{m}}^{a}$/$T_{d5}^{b}$ (^°^C)**	**$I_{\text{p}}^{e}$/*E_g_^f^*/*E_a_^g^*/*E_T_^h^* (eV)**	***S^***i***^***
mCP	408	60/n.d./280	−6.01/3.49/−2.63/3.00	0.08
CBP	485	62/283/413	−5.91/3.44/−2.67/2.60	−0.07
mCPCN	434	97/222/313	−6.08/3.44/−2.64/3.03	0.08
DPEPO	571	n.d./280/322	−6.70/4.00/−2.70/3.30	0.00

*^a^T_g_ and T_m_ were determined using DSC. ^b^T_d5_ was determined using TGA. ^e^I_p_ was determined using PYS. ${}^{\text{f}}{\text{E}}_{\text{g}}$ was taken as the point where the normalized absorption spectra intersected. ^g^E_a_ was calculated using I_p_ and E_g_. ^h^E_T_ was estimated from the onset of the phosphorescent spectra at 5 K. ^i^VASE derived order parameter*.

### Selection of TADF Emitters

We selected three types of pyrimidine-based TADF emitters named **Ac26DPPM** (Nakao et al., 2017), **AcPPM** (Komatsu et al., 2016a), and **PXZPPM** (Komatsu et al., 2016b) with different donor group(s) such as dimethylacridine (Ac) and phenoxiazine (PXZ) (Figure 1 and Table 2). By comparing these three molecules, we can obtain insight into the effects of two factors: (i) donor number, which reflects the molecular shape anisotropy in these comparisons, and (ii) donor structure. Note that all the emitter molecules showed random orientation in neat film (Figure S2). Similar to the host molecules, DFT calculations were performed to evaluate the dipole moment and visualize the ESP and the results are shown in Figure 1 and Figure S1. All the emitters have a small dipole moment <2.0 D, especially **AcPPM** (μ = 0.38 D) and PXZPPM (μ = 0.65 D). The ESP indicates strong negative charge on the π-plane of position 2 of pyrimidine and on the electron-donating end-cap unit(s) of Ac and PXZ. Similar to the host molecules, H atoms on the aromatic rings had positive charge.

**Table 2:** Thermal and optical properties of the TADF emitters.

**Compound**	**Mw (g/mol)**	**$T_{g}^{a}$/$T_{\text{m}}^{a}$/$T_{d5}^{b}$ (^°^C)**	**$I_{\text{p}}^{e}$/*E_g_^f^*/*E_a_^g^*/*E_T_^h^* (eV)**
Ac-26DPPM	516	90/210/383	−5.67/2.90/−2.77/2.80
Ac-PPM	723	n.d./388/442	−5.65/2.80/−2.85/2.65
PXZ-PPM	671	n.d./290/473	−5.65/2.56/−3.09/2.56

*^a^T_g_ and T_m_ were determined using DSC. ^b^T_d5_ was determined using TGA. ^e^I_p_ was determined using PYS. ${}^{\text{f}}{\text{E}}_{\text{g}}$ was taken as the point where the normalized absorption spectra intersected. ^g^E_a_ was calculated using I_p_ and E_g_. ^h^E_T_ was estimated from the onset of the phosphorescent spectra at 5 K*.

### Molecular Orientation of TADF Emitters

**Figure 3:**
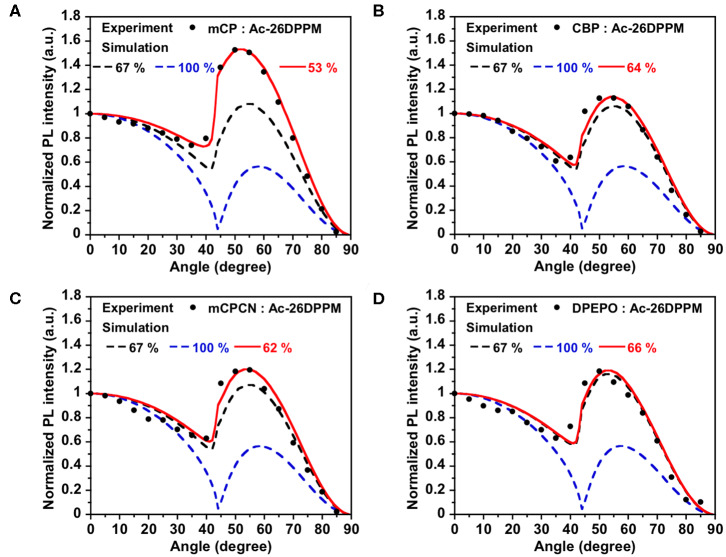
PL intensity of **Ac26DPPM**-doped host films at different angles. The experimental data are in comparison with the fitting curve for different horizontal dipole ratios for **Ac26DPPM** doped in a host film of **(A) mCP**, **(B) CBP**, **(C) mCPCN**, and **(D) DPEPO**.

**Figure 4:**
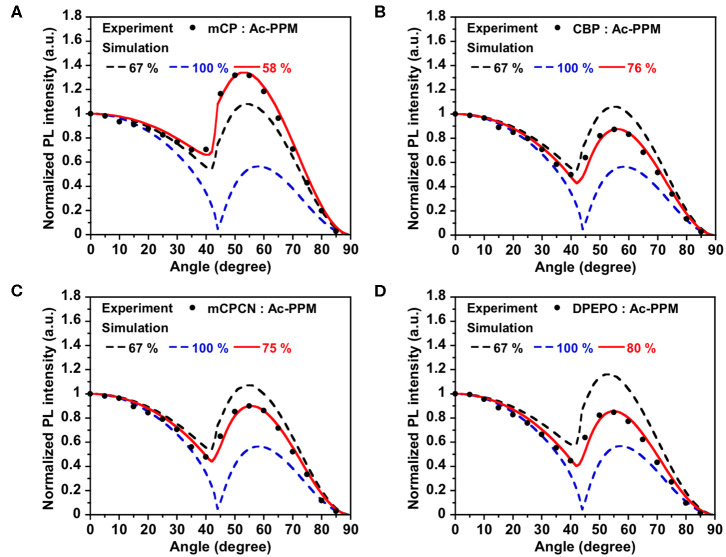
PL intensity of **AcPPM**-doped in **(A) mCP**, **(B) CBP**, **(C) mCPCN**, and **(D) DPEPO** host films at different angles. The experimental data are in comparison with the fitting curve for different horizontal dipole ratios.

**Figure 5:**
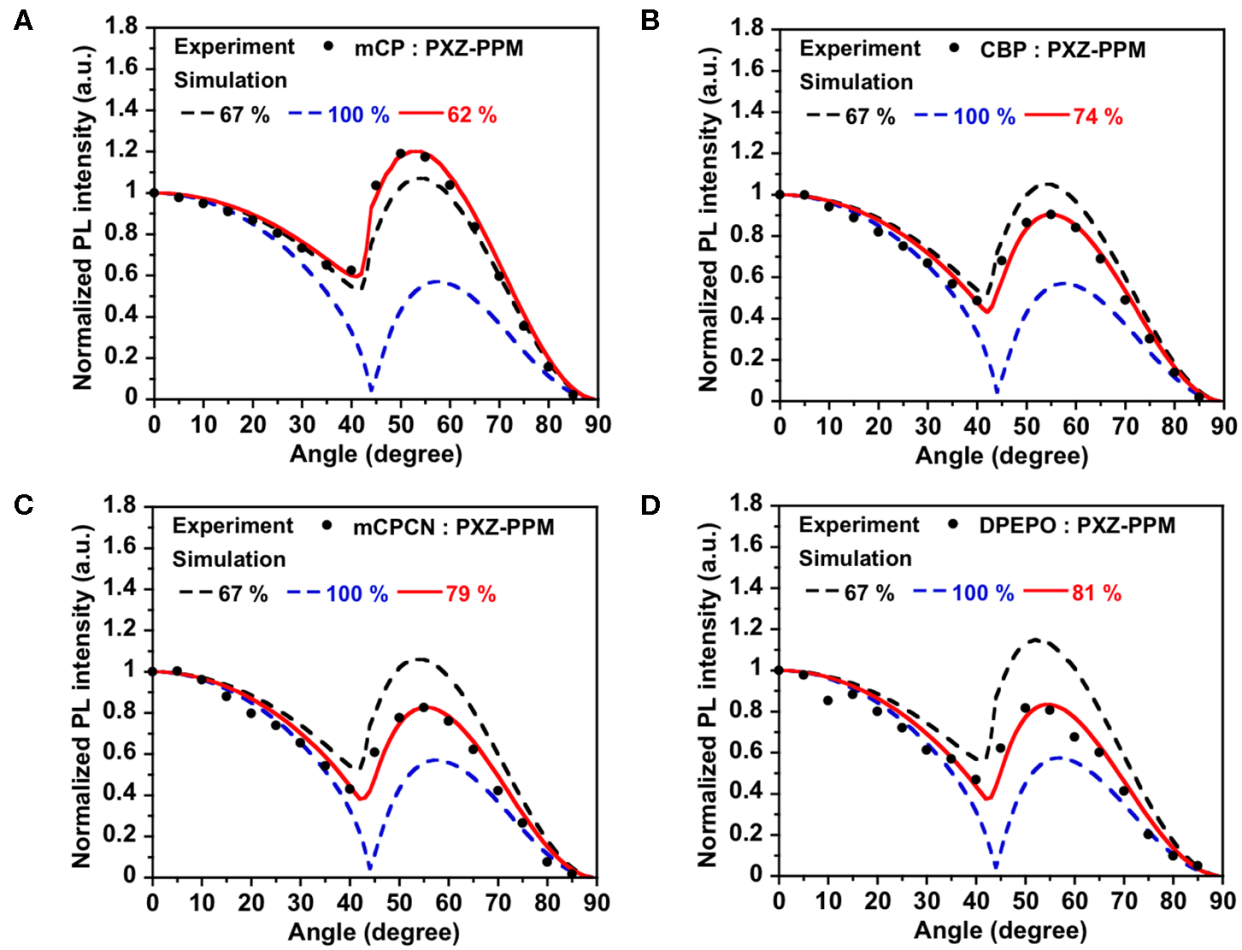
PL intensity of **PXZPPM**-doped in **(A) mCP**, **(B) CBP**, **(C) mCPCN**, and **(D) DPEPO** host films at different angles. The experimental data are in comparison with the fitting curve for different horizontal dipole ratios.

First, we investigated the molecular orientations of 10 wt% **Ac26DPPM**-doped host films by using angle dependent PL measurements. Here, a Θ value of 67% indicates random orientation while a value of 100% indicates perfect horizontal orientation. **Ac26DPPM**-doped **mCP** film showed moderate vertical orientation with Θ = 53%. When **Ac26DPPM** was doped into other host materials (**CBP**, **mCPCN**, and **DPEPO**), all the doped films exhibited almost random orientation with Θ of 62–66% (Figure 3 and Figure S3). Although these films showed random orientation due to the small anisotropy of **Ac26DPPM**, the differences of orientation ratio in the different host materials (ΔΘ) were 9–13%. These differences are not small but meaningfully significant. Similar results were obtained in the case of **AcPPM** (Figure 4 and Figure S4). When host material was changed from **mCP** to one of the other host materials, a significant enhancement of Θ values up to 22% was observed. Since **AcPPM** has larger molecular shape anisotropy than that of **Ac26PMM**, **AcPPM** showed increased horizontal orientation with Θ of 75–80%. Surprisingly, **PXZPPM** also exhibited similar tendencies as the Ac-end-capped emitters (Figure 5 and Figure S5). When **mCP** was used, the **PXZPPM**/**mCP** film showed almost random orientation with Θ of 62%, while when **PXZPPM** was doped into the other host materials, all the doped films exhibited significant horizontal orientation with Θ of 74–81%. Among these emitters, it was determined that bulky methyl groups on Ac end-capping groups do not cause a negative effect toward horizontal orientation as the emitter without methyl groups showed very similar Θ values.

### Underlying Mechanism for Horizontal Orientation

To validate the underlying mechanisms for horizontal orientation, we compared the chemical structures of the host materials. When host was changed from **mCP** to **CBP**, π-conjugation is expanded from phenyl to a biphenyl linker. Therefore, enhanced π-π stacking can be considered as an additional interaction. Note that the dipole moment of **CBP** is calculated to be 0 D, while that of **mCP** is 1.35 D. Further, the dipole moments of the emitters are smaller than 2.0 D. Thus, dipole/dipole interaction can be ruled out as a major interaction. In the case of **mCPCN**, the difference of chemical structure is only a CN group on the carbazole plane. Since the CN group is a strong electron-withdrawing group, the resulting **mCPCN** has a large dipole moment of 6.19 D. However, as mentioned in the case of **CBP**, the contributions from dipole/dipole interaction can be considered to be small because the emitters used in this study have very small dipole moment <2 D. In fact, the ΔΘ of **Ac26DPPM** (ΔΘ = 9%) with a larger dipole moment (μ = 1.89 D) was smaller than that of **AcPPM** (ΔΘ = 17%) with a smaller dipole moment (μ = 0.38 D). Therefore, it is only CH/N weak H-bond of CN group plays a key role. The binding energy of CH/N weak H-bond is 10–20 kJ/mol (Desiraju and Steiner, 1999). This interaction has been reported to be a key to controlling horizontal orientation of oligopyridine-containing electron transporters (Sasabe et al., 2011; Yokoyama et al., 2011; Watanabe et al., 2019a,b). **DPEPO** is a frequently used host material in high efficiency TADF OLEDs, and the films using it as the host showed the largest Θ values among the four host materials. **DPEPO** has a large dipole moment of 8.06 D, and therefore the contributions from dipole/dipole interaction are considered to be large. However, similar to the case of **mCPCN**, the contributions from dipole/dipole interaction can be considered to be small because the emitters used in this study have very small dipole moment <2 D. In fact, the ΔΘ of **Ac26DPPM** (ΔΘ = 13%) with a larger dipole moment (μ = 1.89 D) was smaller than that of **AcPPM** (ΔΘ = 22%) with a smaller dipole moment (μ = 0.38 D). Given that **DPEPO** has short conjugation lengths with steric hindrance, π-π interaction is relatively small. Thus, it can be considered that CH/O weak H-bond from P=O and ether linker plays a key role in determining the horizontal orientation when DPEPO is used as the host material. Recently, Samuel and Zysman-Colman proposed that the significant number of electronegative sites from P=O and ether linker on the surface of **DPEPO** vacuum-deposited film plays an important role for horizontal molecular orientation (Pal et al., 2018). Further, it has also been reported that the strong acceptor property of P=O forms shorter CH···O H-bond, and the bond length of the CH···O H-bond is relatively shorter than that of CH···N H-bond (Desiraju and Steiner, 1999).

Among the three emitters, **PXZPPM** and **AcPPM** showed similar tendencies for horizontal orientation, even though **AcPPM** has four bulky methyl groups on the acridine end-capping units. One possible reason for this is the contributions from multiple CH/π contacts between the methyl groups and the π planes. CH/π contact is a very small interaction, and the binding energy is only 2–3 kJ/mol (Nishio et al., 1998). However, when the molecule has a large number of methyl groups, the contributions would not be negligible in the solid state. In fact, very recently, enhanced horizontal orientation is realized in a series of iridium complexes by introduction of methyl or alkyl groups (Shin et al., 2019). Therefore, we propose that the contributions from multiple CH/π contacts can collaborate with other strong intermolecular interactions to realize significant horizontal orientation.

### OLED Performances

**Figure 6:**
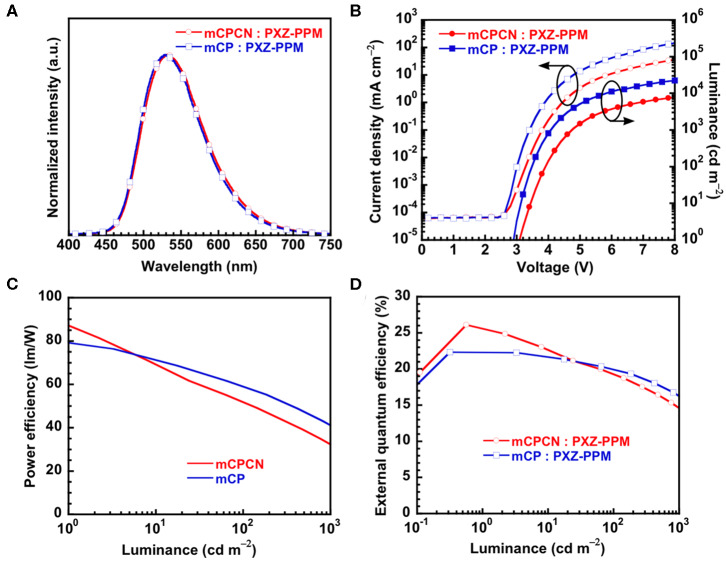
Device performance of **PXZPPM**-based OLEDs; **(A)** EL spectra; **(B)** current density–voltage–luminance characteristics; **(C)** power efficiency–luminance characteristics; **(D)** external quantum efficiency–luminance characteristics.

Finally, we fabricated OLEDs using **PXZPPM**/hosts as an emission layer to show the effect of horizontal orientation on the OLED efficiency. Here, we used two types of hosts, **mCP** (Θ = 62%) and **mCPCN** (Θ = 79%). Note that we did not use DPEPO as a host material because the chemical structure is totally different from mCP. The structure of the OLED was [indium tin oxide (ITO) anode (130 nm)/triphenylamine-containing polymer: 4-isopropyl-4′-methyldiphenyliodonium tetrakis(pentafluorophenyl)borate (**PPBI**) (20 nm) (Kido et al., 1997)/di-[4-(N,N-ditolyl-amino)-phenyl]cyclohexane (**TAPC**) (25 nm)/4,4′,4″-tris(N-carbazolyl)triphenylamine (**TCTA**) (5 nm)/10 wt% **PXZPPM**-doped **mCP**, or **mCPCN** (10 nm)/3,3″,5,5′-tetra(3-pyridyl)-1,1′;3′,1″-terphenyl (**B3PyPB**) (50 nm) (Sasabe et al., 2008)/LiF (0.5 nm)/Al cathode (100 nm)]. **TAPC** and **TCTA** were used as the hole transport layers, **B3PyPB** as the electron transport layer, and LiF as the electron injection layer. The chemical structures of these materials are shown in Figure S6. Figure 6 shows the electroluminescence (EL) spectra, the current density (J)–voltage (V)–luminance (L), and η_ext_-L characteristics. The electroluminescent characteristics are summarized in Table 3. The EL spectra of both devices showed similar emission with peaks at 529 nm for **mCP** and at 531 nm for **mCPCN** host materials. The maximum η_ext_ values were recorded to be 22.3% for **mCP** and 26.1% for **mCPCN**. Moreover, the light distribution patterns evaluated by Lambertian factor of these devices were similar, and recorded to be 0.99 for **mCP**, and 0.97 for **mCPCN**, as shown in Figure S6. The photoluminescent quantum yields (η_PL_) of **PXZPPM**/host films were similar for **mCP** (71%) and **mCPCN** (69%) hosts. Given that the carrier balance factor is similar at the peak efficiency, the differences in the maximum η_ext_ values can be attributed to the differences in Θ values of the different host materials. Note that we also fabricated the devices using **CBP** and **DPEPO** as a host material. As a result, higher η_PL_ and Θ values gave superior device performances (Figure S7 and Table S1). Enhancing the horizontal orientation of the emitter apparently increases the light coupling efficiency, thus resulting in a higher η_ext_ value.

**Table 3:** Summary of **PXZPPM**-based OLED performance.

**Host**	**V_**on**_*^**a**^* (V)**	**V_**100**_/η_**c, 100**_/η_**p, 100**_/η_**ext, 100**_*^***b***^* (V/cd A^**−1**^/lm W^**−1**^/%)**	**V_**1000**_/η_**c, 1000**_/η_**p, 1000**_/η_**ext, 1000**_*^***c***^* (V/cd A^**−1**^/lm W^**−1**^/%)**	**η_**c, max**_/η_**p, max**_/η_**ext, max**_*^***d***^* (cd A^**−1**^/lm W^**−1**^/%)**
mCP	2.85	3.46/65.7/59.7/20.0	4.07/53.5/41.4/16.3	73.1/82.1/22.3
mCPCN	3.05	3.89/64.8/52.4/19.4	4.74/48.9/32.5/14.6	87.2/91.4/26.1

*^a^Turn-on voltage at 1 cd m^−2^. ^b^Voltage (V), current efficiency (η_c_), power efficiency (η_p_), and external quantum efficiency (η_ext_) at 100 cd m^−2^. ^c^V, η_c_, η_p_, and η_ext_ at 1,000 cd m^−2^. ${}^{\text{d}}\eta_{\text{c}}$, η_p_, and η_ext_ at maximum*.

## Conclusion

We performed a systematic investigation on the molecular orientations of TADF emitters in a series of carbazole-based host materials. TADF emitters used in this study have a small dipole moment <2 D. To validate the underlying mechanisms for horizontal orientation, we changed the chemical structure of **mCP** by: (i) extending the π-conjugation to form **CBP**, and (ii) the introduction of a CN substituent to form **mCPCN**. In addition, a phosphine-oxide host material, **DPEPO**, was used. Although all the emitters and the host materials had random orientation as a neat film, the emitters with larger molecular shape anisotropy, **AcPPM** and **PXZPPM**, showed enhanced horizontal orientation up to Θ ~ 81% in all host films except **mCP**. The third emitter, **Ac26DPPM**, has a smaller molecular shape anisotropy, and subsequently showed vertical orientation in the **mCP** host film (Θ = 53%). However, when doped into the other host material, **Ac26DPPM** showed random orientation (Θ ~ 66%). From these results, it can be considered that (i) an increase in the π-conjugation leads to stronger π-π stacking, (ii) the introduction of CN group induces CH/N weak H-bonds, and (iii) the introduction of a strong acceptor P=O and ether groups induces CH/O H-bonds, and all these interactions enhance the horizontal orientation of TADF emitters. In addition, we propose that the contributions from multiple CH/π contacts can collaborate with other strong intermolecular interactions such as π-π stacking, CH/N and CH/O H-bonds to realize significant horizontal orientation. Finally, we fabricated an **PXZPPM**-based OLED that achieved an η_ext_ of 26% using an EML with Θ of 79%, which is higher than that of an almost randomly oriented EML with Θ of 62% (η_ext_ = 22%). We believe that our results will be beneficial in revealing the underlying mechanism for horizontal orientation leading to superior OLED performances.

## Data Availability Statement

All datasets generated for this study are included in the article/Supplementary Material.

## Author Contributions

HS and SO conceived the project. HS, YC, SO, and DY interpreted the data and prepared the manuscript and supplementary materials. HS, SO, and JK supervised the project. YC, SO, and DY designed experiments. HA, TO, RK, and YW synthesized and characterized materials. YC, YW, and DY prepared samples, performed variable-angle spectroscopic ellipsometry measurements, and related data analysis. YC, SO, and HA prepared samples and performed angle dependent PL measurements. YC and HA fabricated devices. All authors discussed the results and commented on the manuscript.

## Conflict of Interest

The authors declare that the research was conducted in the absence of any commercial or financial relationships that could be construed as a potential conflict of interest.

## References

[B1] Adachi C. (2014). Third-generation organic electroluminescence materials. Jpn. J. Appl. Phys. 53:60101. 10.7567/JJAP.53.06010130141908

[B2] Ahn D. H. Kim S. W. Lee H. Ko I. J. Karthik D. Lee J. Y. (2019). Highly efficient blue thermally activated delayed fluorescence emitters based on symmetrical and rigid oxygen-bridged boron acceptors. Nat. Photonics 13, 540–546. 10.1038/s41566-019-0415-5

[B3] Desiraju G. R. Steiner T. (1999). The Weak Hydrogen Bond–In Structural Chemistry and Biology. New York, NY: Oxford University Press.

[B4] Friederich P. Coehoorn R. Wenzel W. (2017). Molecular origin of the anisotropic dye orientation in emissive layers of organic light emitting diodes. Chem. Mater. 29:9528 10.1021/acs.chemmater.7b03742

[B5] Frischeisen J. Yokoyama D. Endo A. Adachi C. Brutting W. (2011). Increased light outcoupling efficiency in dye-doped small molecule organic light-emitting diodes with horizontally oriented emitters. Org. Electron. 12, 809–817. 10.1016/j.orgel.2011.02.005

[B6] Gujral A. Yu L. Ediger M. D. (2018). Anisotropic organic glasses. Curr. Opin. Solid State Mater. Sci. 22, 49–57. 10.1016/j.cossms.2017.11.001

[B7] Han C. M. Zhao Y. B. Xu H. Chen J. S. Deng Z. P. Ma D. G.. (2011). A simple phosphine-oxide host with a multi-insulating structure: high triplet energy level for efficient blue electrophosphorescence. Chem. Eur. J. 17, 5800–5803. 10.1002/chem.201100254 21503995

[B8] Im Y. Kim M. Cho Y. J. Seo J. A. Yook K. S. Lee J. Y. (2017). Molecular design strategy of organic thermally activated delayed fluorescence emitters. Chem. Mater. 29, 1946–1963. 10.1021/acs.chemmater.6b05324

[B9] Kaji H. Suzuki H. Fukushima T. Shizu K. Suzuki K. Kubo S.. (2015). Purely organic electroluminescent material realizing 100% conversion from electricity to light. Nat. Commun. 6:8476. 10.1038/ncomms9476 26477390 PMC4634127

[B10] Kido J. Harada G. Komada M. Shionoya H. Nagai K. (1997). Aromatic-amine-containing polymers for organic electroluminescent devices. ACS Symposium Ser. 672, 381–394. 10.1021/bk-1997-0672.ch025

[B11] Kim K. H. Kim J. J. (2018). Origin and control of orientation of phosphorescent and TADF dyes for high-efficiency OLEDs. Adv. Mater. 30:1705600. 10.1002/adma.201705600 29707823

[B12] Komatsu R. Sasabe H. Nakao K. Hayasaka Y. Ohsawa T. Kido J. (2016a). Unlocking the potential of pyrimidine conjugate emitters to realize high-performance organic light-emitting devices. Adv. Opt. Mater. 5. 10.1002/adom.20160067528121118

[B13] Komatsu R. Sasabe H. Seino Y. Nakao K. Kido J. (2016b). Light-blue thermally activated delayed fluorescent emitters realizing a high external quantum efficiency of 25% and unprecedented low drive voltages in OLEDs. J. Mater. Chem. C 4:2274. 10.1039/C5TC04057D31069215

[B14] Komatsu R. Sasabe H. Kido J. (2018). Recent progress of pyrimidine derivatives for high-performance organic light-emitting devices. J. Photon. Energy 8:032108 10.1117/1.JPE.8.032108

[B15] Komino T. Sagara Y. Tanaka H. Oki Y. Nakamura N. Fujimoto H. (2016). Electroluminescence from completely horizontally oriented dye molecules. Appl. Phys. Lett. 108:241106 10.1063/1.4954163

[B16] Kondo Y. Yoshiura K. Kitera S. Nishi H. Oda S. Gotoh H. (2019). Narrowband deep-blue organic light-emitting diode featuring an organoboron-based emitter. Nat. Photonics 13, 678–682. 10.1038/s41566-019-0476-5

[B17] Lee Y. T. Tseng P. C. Komino T. Mamada M. Ortiz R. J. Leung M. K.. (2018). Simple molecular-engineering approach for enhancing orientation and outcoupling efficiency of thermally activated delayed fluorescent emitters without red-shifting emission. ACS Appl. Mater. Interfaces 10, 43842–43849. 10.1021/acsami.8b16199 30484304

[B18] Lin M. S. Yang S. J. Chang H. W. Huang Y. H. Tsai Y. T. Wu C. C. (2012). Incorporation of a CN group into mCP: a new bipolar host material for highly efficient blue and white electrophosphorescent devices. J. Mater. Chem. 22, 16114–16120. 10.1039/c2jm32717a

[B19] Lin T. A. Chatterjee T. Tsai W. L. Lee W. K. Wu M. J. Jiao M.. (2016). Sky-blue organic light emitting diode with 37% external quantum efficiency using thermally activated delayed fluorescence from spiroacridine-triazine hybrid. Adv. Mater. 28, 6976–6983. 10.1002/adma.201601675 27271917

[B20] Liu M. Komatsu R. Cai X. Hotta K. Sato S. Liu K. (2017). Horizontally orientated sticklike emitters: enhancement of intrinsic out-coupling factor and electroluminescence performance. Chem. Mater. 29, 8630–8636. 10.1021/acs.chemmater.7b02403

[B21] Mayr C. Brütting W. (2015). Control of molecular dye orientation in organic luminescent films by the glass transition temperature of the host material. Chem. Mater. 27, 2759–2762. 10.1021/acs.chemmater.5b00062

[B22] Moon C.-K. Kim K.-H. Lee J. W. Kim J.-J. (2015). Influence of host molecules on emitting dipole orientation of phosphorescent iridium complexes. Chem. Mater. 27, 2767–2769. 10.1021/acs.chemmater.5b00469

[B23] Nakao K. Sasabe H. Komatsu R. Hayasaka Y. Ohsawa T. Kido J. (2017). Significant enhancement of blue OLED performances through molecular engineering of pyrimidine-based emitter. Adv. Opt. Mater. 5:1600843 10.1002/adom.201600843

[B24] Nishio M. Hirota M. Umezawa Y. (1998). The CH/π Interaction–Evidence, Nature, and Consequences. New York, NY: Wiley-VCH.

[B25] Pal A. K. Krotkus S. Fontani M. Mackenzie C. F. R. Cordes D. B. Slawin A. M. Z. (2018). High-efficiency deep-blue-emitting organic light-emitting diodes based on iridium(III) carbene complexes. Adv. Mater. 30:e1804231 10.1002/adma.201804231 30318632

[B26] Rajamalli P. Senthilkumar N. Huang P. Y. Ren-Wu C. C. Lin H. W. Cheng C. H. (2017). New molecular design concurrently providing superior pure blue, thermally activated delayed fluorescence and optical out-coupling efficiencies. J. Am. Chem. Soc. 139, 10948–10951. 10.1021/jacs.7b03848 28745874

[B27] Sasabe H. Gonmori E. Chiba T. Li Y. J. Tanaka D. Su S. J. (2008). Wide-energy-gap electron-transport materials containing 3,5-dipyridylphenyl moieties for an ultra high efficiency blue organic light-emitting device. Chem. Mater. 20, 5951–5953. 10.1021/cm801727d

[B28] Sasabe H. Kido J. (2013). Development of high performance OLEDs for general lighting. J. Mater. Chem. C 1:1699 10.1039/c2tc00584k

[B29] Sasabe H. Tanaka D. Yokoyama D. Chiba T. Pu Y.-J. Nakayama K.-I. (2011). Influence of substituted pyridine rings on physical properties and electron mobilities of 2-methylpyrimidine skeleton-based electron transporters. Adv. Func. Mater. 21, 336–342. 10.1002/adfm.201001252

[B30] Schmidt T. D. Lampe T. Sylvinson D. M. R. Djurovich P. I. Thompson M. E. Brutting W. (2017). Emitter orientation as a key parameter in organic light-emitting diodes. Phys. Rev. Appl. 8:037001 10.1103/PhysRevApplied.8.037001

[B31] Shibata M. Sakai Y. Yokoyama D. (2015). Advantages and disadvantages of vacuum-deposited and spin-coated amorphous organic semiconductor films for organic light-emitting diodes. J. Mater. Chem. C 3:11178 10.1039/C5TC01911G

[B32] Shin H. Ha Y. H. Kim H. G. Kim R. Kwon S. K. Kim Y. H.. (2019). Controlling horizontal dipole orientation and emission spectrum of Ir complexes by chemical design of ancillary ligands for efficient deep-blue organic light-emitting diodes. Adv. Mater. 31:1808102. 10.1002/adma.201808102 30972824

[B33] Uoyama H. Goushi K. Shizu K. Nomura H. Adachi C. (2012). Highly efficient organic light-emitting diodes from delayed fluorescence. Nature 492, 234–238. 10.1038/nature11687 23235877

[B34] Watanabe Y. Sasabe H. Kido J. (2019a). Review of molecular engineering for horizontal molecular orientation in organic light-emitting devices. Bull. Chem. Soc. Jpn. 92, 716–728. 10.1246/bcsj.20180336

[B35] Watanabe Y. Yokoyama D. Koganezawa T. Katagiri H. Ito T. Ohisa S.. (2019b). Control of molecular orientation in organic semiconductor films using weak hydrogen bonds. Adv. Mater. 31:1808300. 10.1002/adma.201808300 30848005

[B36] Wong M. Y. Zysman-Colman E. (2017). Purely organic thermally activated delayed fluorescence materials for organic light-emitting diodes. Adv. Mater. 29:1605444. 10.1002/adma.201605444 28256751

[B37] Wu T.-L. Huang M.-J. Lin C.-C. Huang P.-Y. Chou T.-Y. Chen-Cheng R.-W. (2018). Diboron compound-based organic light-emitting diodes with high efficiency and reduced efficiency roll-off. Nat. Photonics 12, 235–240. 10.1038/s41566-018-0112-9

[B38] Yang Z. Y. Mao Z. Xie Z. L. Zhang Y. Liu S. W. Zhao J.. (2017). Recent advances in organic thermally activated delayed fluorescence materials. Chem. Soc. Rev. 46, 915–1016. 10.1039/C6CS00368K 28117864

[B39] Yokoyama D. (2011). Molecular orientation in small-molecule organic light-emitting diodes. J. Mater. Chem. 21:19187 10.1039/c1jm13417e

[B40] Yokoyama D. Sakaguchi A. Suzuki M. Adachi C. (2009). Horizontal orientation of linear-shaped organic molecules having bulky substituents in neat and doped vacuum-deposited amorphous films. Org. Electron. 10, 127–137. 10.1016/j.orgel.2008.10.010

[B41] Yokoyama D. Sasabe H. Furukawa Y. Adachi C. Kido J. (2011). Molecular stacking induced by intermolecular C-H···N hydrogen bonds leading to high carrier mobility in vacuum-deposited organic films. Adv. Func. Mater. 21, 1375–1382. 10.1002/adfm.201001919

