# Supplementary material for: Molecular Orientations of Delayed Fluorescent Emitters in a Series of Carbazole-Based Host Materials
Source: Front Chem. 2020 May 25;8:427. doi: 10.3389/fchem.2020.00427 (PMC7262557; doi:10.3389/fchem.2020.00427)
Supplement: Supplementary file 1 [file Data_Sheet_1.pdf]

Supplementary Material **for:**

Molecular Orientations of Delayed Fluorescent Emitters in a Series of Carbazole-based Host Materials

Hisahiro Sasabe[1,2,3]*, Yuki Chikayasu[1], Satoru Ohisa[1,2,3], Hiroki Arai[1], Tatsuya Ohsawa[1], Ryutaro Komatsu[1], Yuichiro Watanabe[1], Daisuke Yokoyama[1,3], Junji Kido[1,2,3]*

[1]Department of Organic Materials Science, Graduate School of Organic Materials Science, Yamagata University, 4-3-16 Jonan, Yonezawa, Yamagata 992-8510, Japan

[2]Frontier Center for Organic Materials (FROM) Yamagata University

[3]Research Center for Organic Electronics (ROEL) Yamagata University

Experimental

General Considerations: **mCPCN**[1], **DPEPO**[2], **AcPPM**[3], **Ac26DPPM**[4], and **PXZPPM**[5] were prepared according to the literature procedure. **mCP** and **CBP** was purchased from TCI. All organics were used after train sublimation. DSC was performed using a Perkin-Elmer Diamond DSC Pyris instrument under nitrogen atmosphere at a heating rate of 10 ˚C min^{-1}. TGA was undertaken using a SEIKO EXSTAR 6000 TG/DTA 6200 unit under nitrogen atmosphere at a heating rate of 10 ˚C min^{-1}. UV–vis spectra was measured using a Shimadzu UV-3150 UV–vis–NIR spectrophotometer. Photoluminescence spectra were measured using a FluoroMax-2 (Jobin-Yvon-Spex) luminescence spectrometer. PL quantum efficiencies were measured on a Hamamatsu photonics C9920-01 integral sphere system. All quantum chemistry calculations were performed using the Gaussian 09 suite of programs[6]. The I_p was determined by a PYS under the vacuum (=10^{-3} Pa). The experiments of VASE were performed by using a fast spectroscopic ellipsometer (M-2000U, J. A. Woollam Co., Inc.). Measurements were taken at seven multiple angles of the incident light from 45° to 75° in a step of 5°. The analysis of the data was performed via using the software "WVASE32" (J. A. Woollam Co., Inc.), which can perform an analysis of all the data at all the different incident angles and wavelengths. Doped films of emitters in hosts with 30 nm thickness were prepared for angle-dependent photoluminescence intensities experiment. The samples were fixed on a stage with adjustable axis and attached to a cylindrical lens. The wavelength of the excited light was set to 340 nm, and the PL intensities at different adjusted angles were via a p-wave polarizer and collected by a Konica Minolta CS-2000.

Device Fabrication and Characterization: The substrates were cleaned with ultrapurified water and organic solvents, and then dry-cleaned for 30 minutes by exposure to UV–ozone. The organic layers were deposited onto the ITO substrates under the vacuum (=10^{-5} Pa), successively. Al was patterned using a shadow mask with an array of 2 mm × 2 mm openings

without breaking the vacuum (=10^{-5} Pa). The electroluminescent (EL) were taken using an optical multichannel analyzer Hamamatsu Photonics PMA-11. The current density–voltage and luminance–voltage characteristics were measured by using a Keithley source measure unit 2400 and a Minolta CS200 luminance meter, respectively.

References

[1] M.-S. Lin, S.-J. Yang, H.-W. Chang, Y.-H. Huang, Y.-T. Tsai, C.-C. Wu, S.-H. Chou, E. Mondal, K.-T. Wong, *J. Mater. Chem.* **2012**, *22*, 16114.

[2] C. Han, Y. Zhao, H. Xu, J. Chen, Z. Deng, D. Ma, Q. Li, P. Yan, *Chem. Eur. J.* **2011**, *17*, 5800.

[3] R. Komatsu, H. Sasabe, Y. Seino, K Nakao, J. Kido, *J. Mater. Chem. C* **2016**, 4, 2274–2278.

[4] K. Nakao, H. Sasabe, R. Komatsu, Y. Hayasaka, T. Osawa, J. Kido, *Adv. Opt. Mater.* **2017**, 5, 1600843.

[5] R. Komatsu, H. Sasabe, K. Nakao, Y. Hayasaka, T. Osawa, J. Kido, *Adv. Opt. Mater.* **2017**, *5*, 1600675.

[6] *Gaussian 09*, Revision D.01, M. J. Frisch, G. W. Trucks, H. B. Schlegel, G. E. Scuseria, M. A. Robb, J. R. Cheeseman, G. Scalmani, V. Barone, B. Mennucci, G. A. Petersson, H. Nakatsuji, M. Caricato, X. Li, H. P. Hratchian, A. F. Izmaylov, J. Bloino, G. Zheng, J. L. Sonnenberg, M. Hada, M. Ehara, K. Toyota, R. Fukuda, J. Hasegawa, M. Ishida, T. Nakajima, Y. Honda, O. Kitao, H. Nakai, T. Vreven, J. A. Montgomery, Jr., J. E. Peralta, F. Ogliaro, M. Bearpark, J. J. Heyd, E. Brothers, K. N. Kudin, V. N. Staroverov, R. Kobayashi, J. Normand, K. Raghavachari, A. Rendell, J. C. Burant, S. S. Iyengar, J. Tomasi, M. Cossi, N. Rega, J. M. Millam, M. Klene, J. E. Knox, J. B. Cross, V. Bakken, C. Adamo, J. Jaramillo, R. Gomperts, R. E. Stratmann, O. Yazyev, A. J. Austin, R. Cammi, C. Pomelli, J. W. Ochterski, R. L. Martin, K. Morokuma, V. G. Zakrzewski, G. A. Voth, P. Salvador, J. J. Dannenberg, S. Dapprich, A. D. Daniels, Ö. Farkas, J. B. Foresman, J. V. Ortiz, J. Cioslowski, and D. J. Fox, Gaussian, Inc., Wallingford CT, 2013.

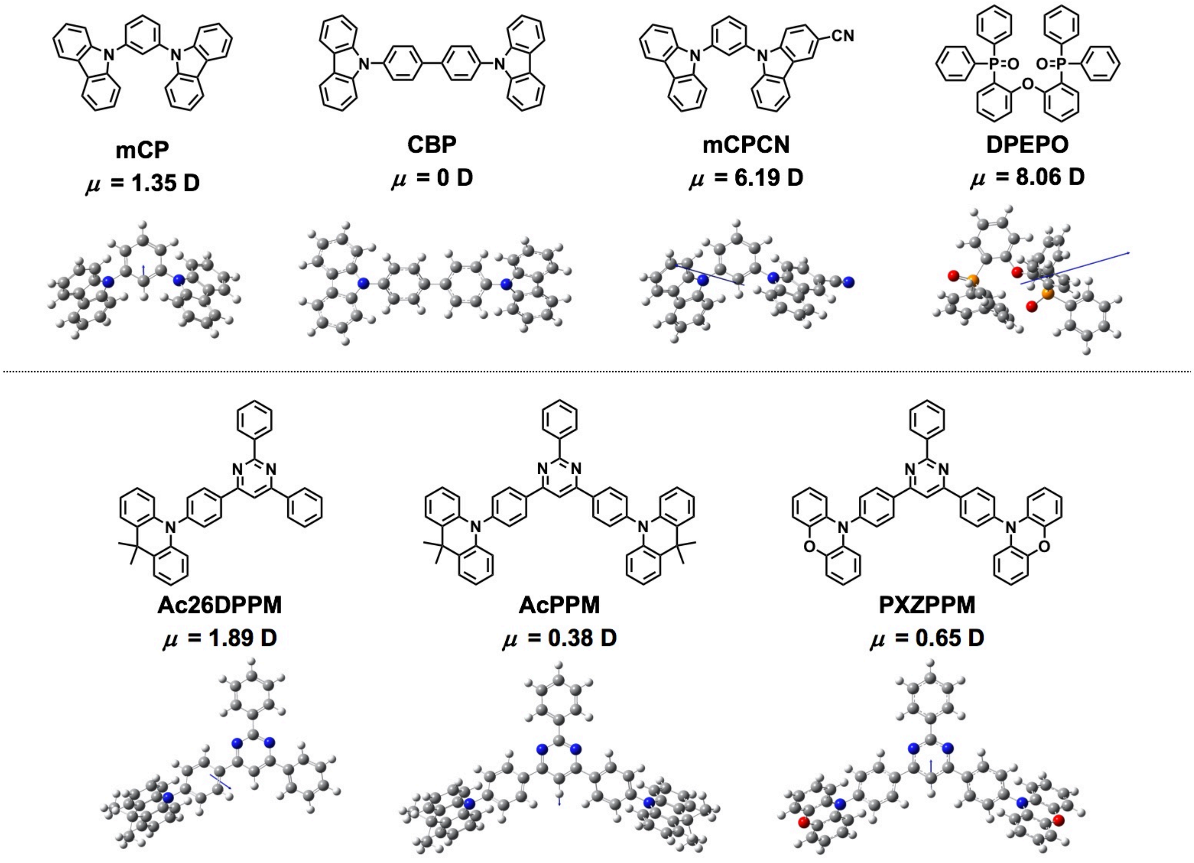

Figure S1. Chemical structures, values of dipole moments, and the directions of dipole moments of the host molecules (top) and the TADF emitters (bottom) used in this study.

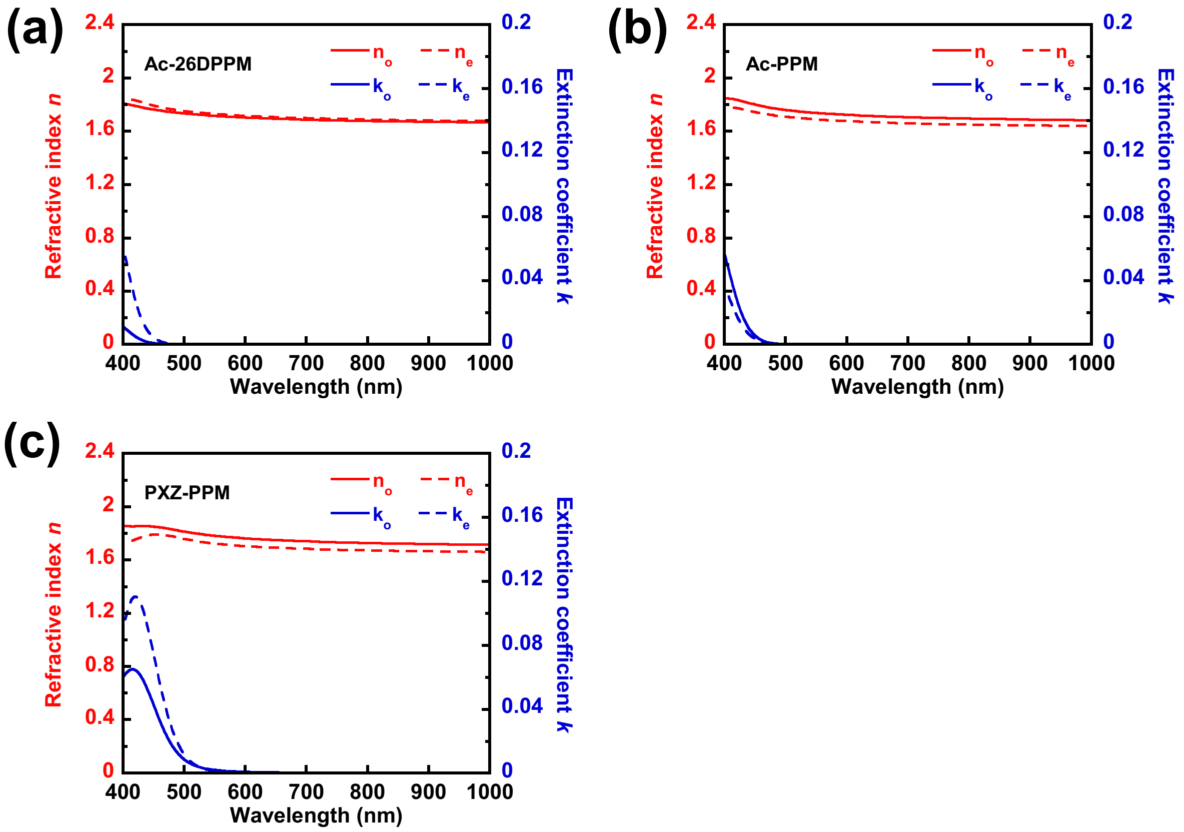

Figure S2. Ordinary (horizontal direction) and extraordinary (vertical direction) refractive indices and extinction coefficients obtained from neat films of (a) **Ac26DPPM**, (b) **AcPPM**, and (c) **PXZPPM**.

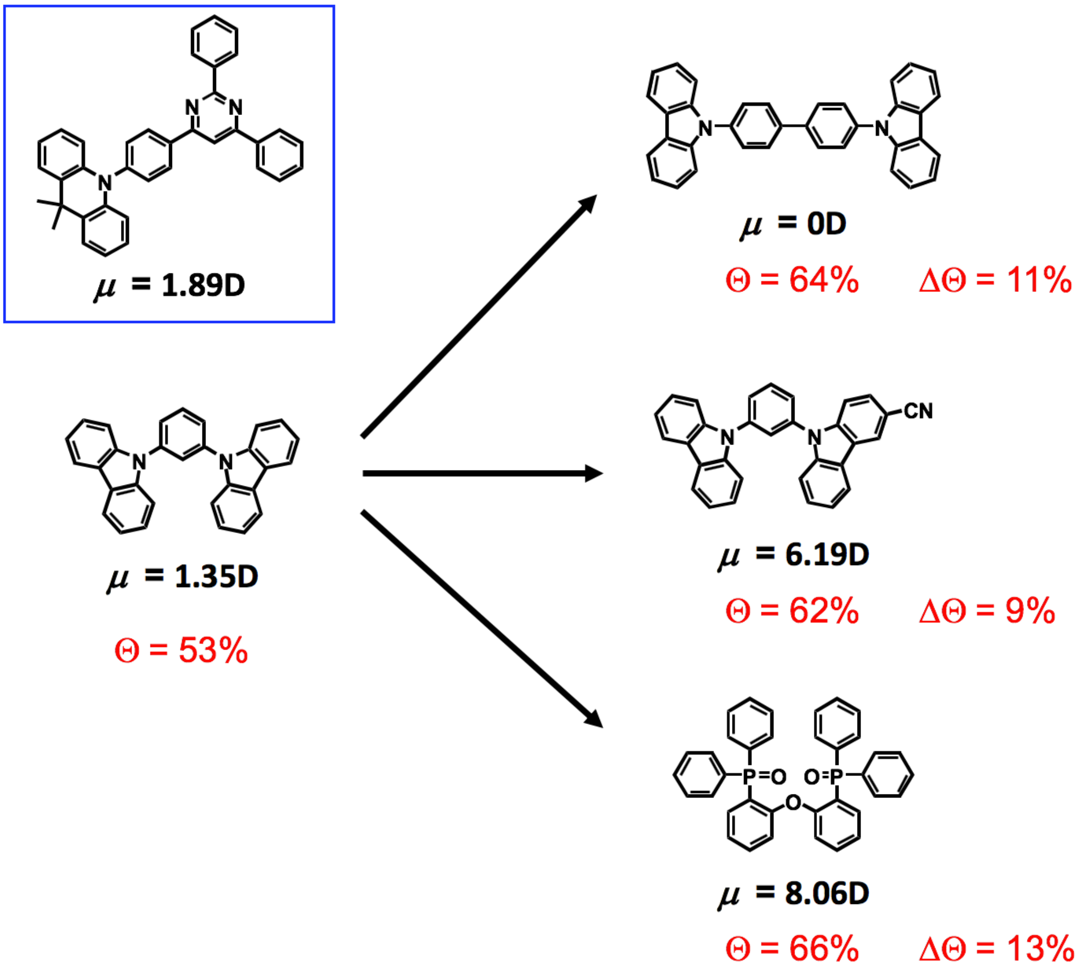

Figure S3. Chemical structures of **Ac26DPPM** and host molecules with the dipole moments, horizontal orientation ratios (Θ), and the difference of Θ values (ΔΘ) compared to **Ac26DPPM** doped into mCP.

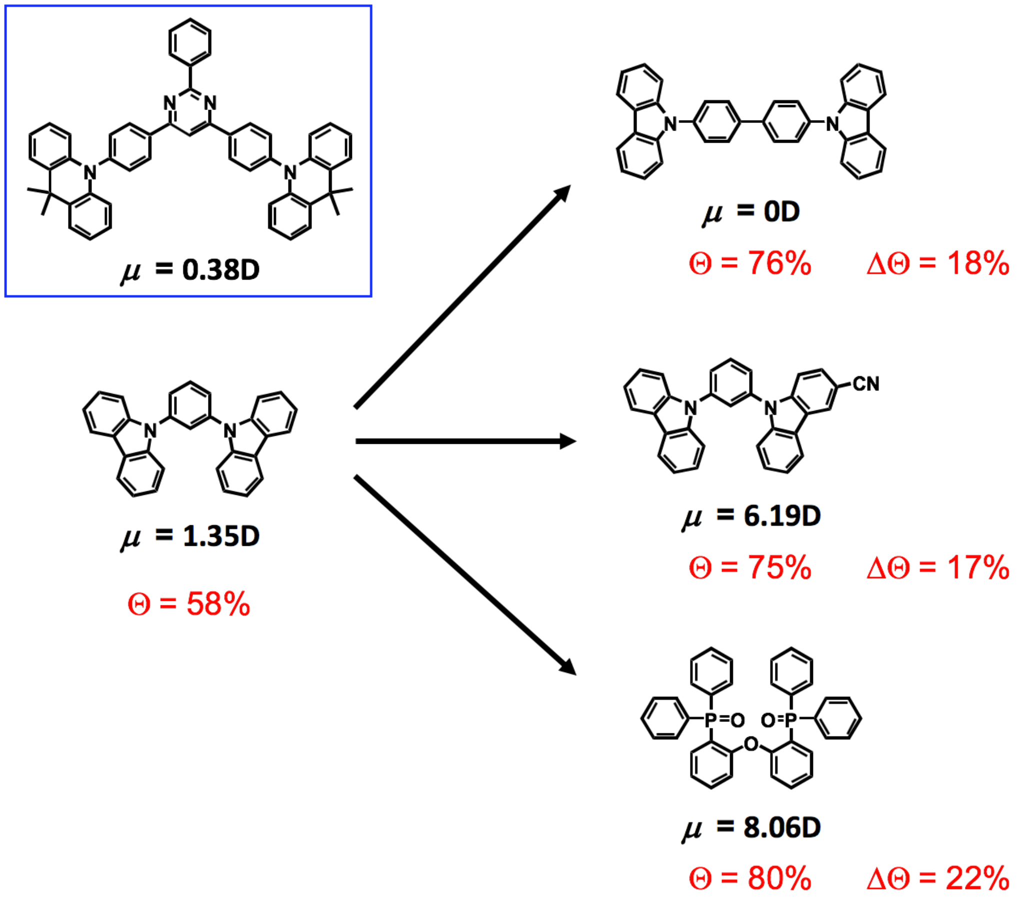

Figure S4. Chemical structures of **AcPPM** and host molecules with the dipole moments, horizontal orientation ratios (Θ), and the difference of Θ values (ΔΘ) compared to **AcPPM** doped into mCP.

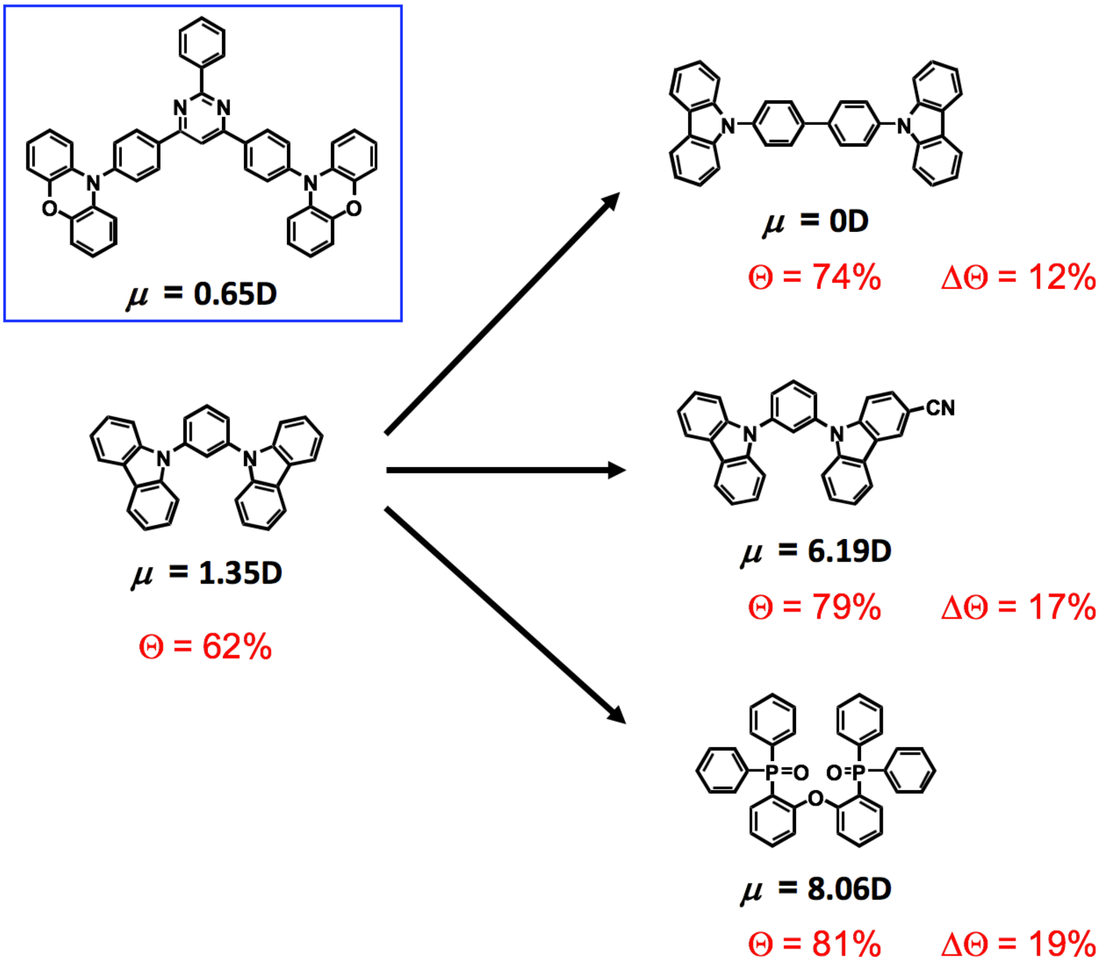

Figure S5. Chemical structures of **PXZPPM** and the host molecules with dipole moments, horizontal orientation ratios (Θ), and the difference of Θ values (ΔΘ) compared to **PXZPPM** doped into mCP.

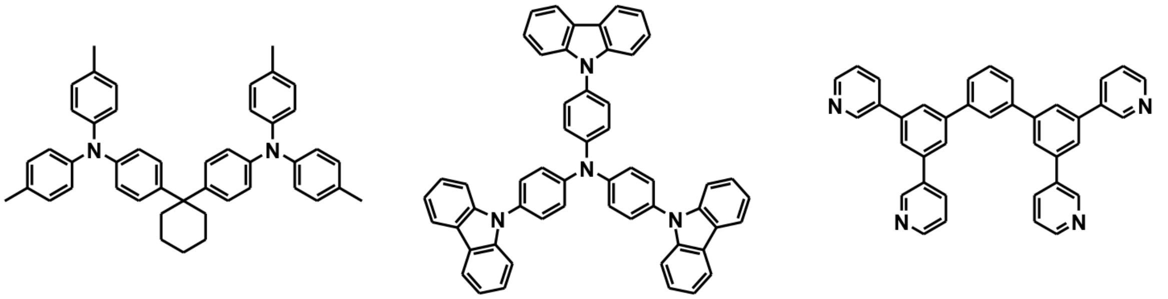

TAPC **TCTA** **B3PyPB**

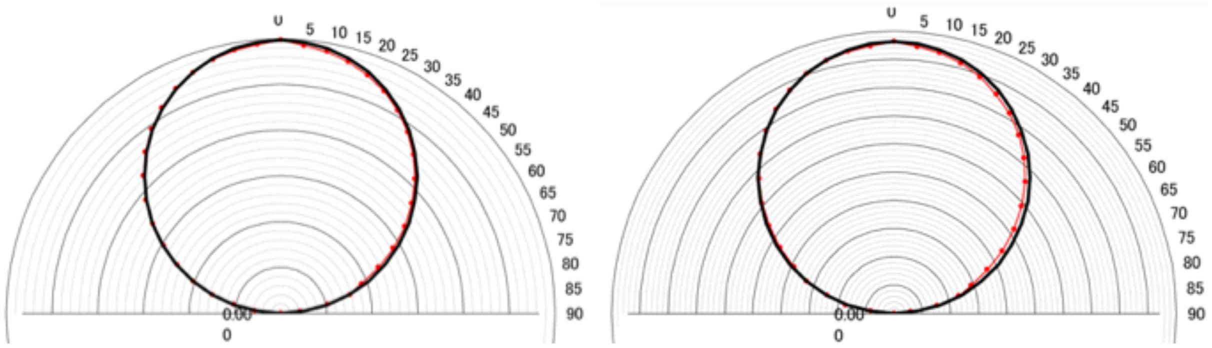

mCP : PXZ-PPM **mCPCN : PXZ-PPM**

Figure S6. Chemical structures of materials used in the OLEDs (top), and the light distribution patterns of each device (bottom).

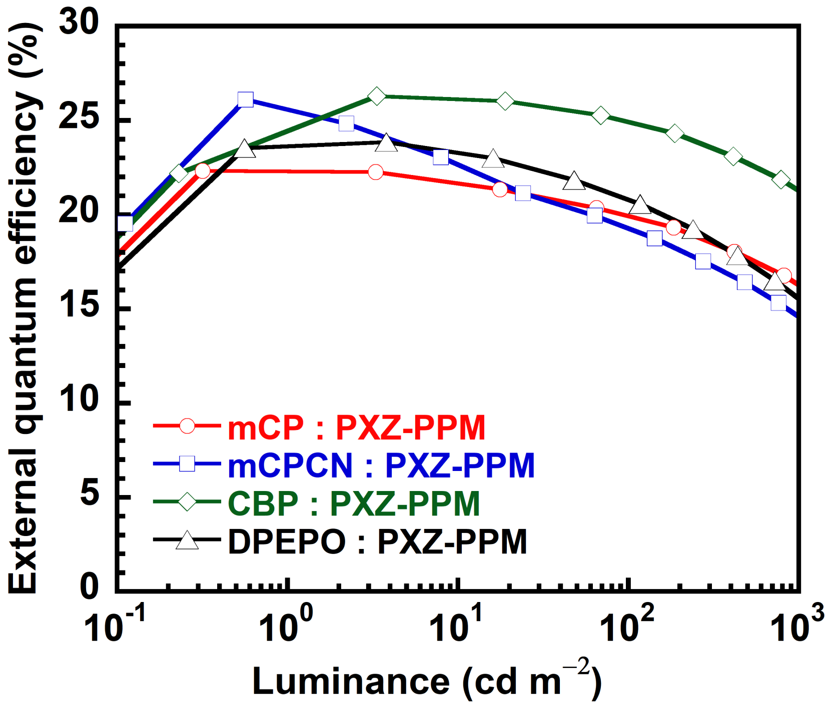

Figure S7. External quantum efficiency–luminance characteristics of **PXZPPM**-based OLEDs with different host materials.

Table S1. Summary of **PXZPPM**-based OLED performance.

Host	η_{PL}[a] (%)	Θ[b] (%)	V_{on}[c] (V)	$V_{100}/\eta_{c,100}/\eta_{p,100}/\eta_{ext,100}$[d] (V/cd A^{-1}/lm W^{-1}/%)	$V_{1000}/\eta_{c,1000}/\eta_{p,1000}/\eta_{ext,1000}$[e] (V/cd A^{-1}/lm W^{-1}/%)	$\eta_{c,max}/\eta_{p,max}/\eta_{ext,max}$[f] (cd A^{-1}/lm W^{-1}/%)
mCP	71	62	2.85	3.46/65.7/59.7/20.0	4.07/53.5/41.4/16.3	73.1/82.1/22.3
mCPCN	69	79	3.05	3.89/64.8/52.4/19.4	4.74/48.9/32.5/14.6	87.2/91.4/26.1
CBP	71	74	2.85	3.45/84.0/76.5/25.0	4.08/71.7/55.2/21.4	88.3/92.4/26.3
DPEPO	57	81	2.63	3.35/69.6/65.1/20.9	4.15/51.7/39.2/15.6	79.2/94.5/23.8

[a]Photoluminescent quantum yield (η_{PL}) of 10wt% **PXZPPM**-doped host film. [b]Horizontal orientation ratio (Θ) of 10wt% **PXZPPM**-doped host film. [c]Turn-on voltage at 1 cd m^{-2}. [d]Voltage (V), current efficiency (η_c), power efficiency (η_p), and external quantum efficiency (η_{ext}) at 100 cd m^{-2}. [e]V, η_c, η_p, and η_{ext} at 1000 cd m^{-2}. [f]η_c, η_p, and η_{ext} at maximum.
